# Antenatal Steroid Utilization in Ethiopia

**DOI:** 10.1177/2333794X21990344

**Published:** 2021-02-05

**Authors:** Gesit Metaferia, Mahlet Abayneh, Sara Aynalem, Abayneh G. Demisse, Asrat G. Demtse, Beza Eshetu, Amha Mekasha, Bogale Worku, Assaye K. Nigussie, Elizabeth M. McClure, Robert L. Goldenberg, Lulu M. Muhe

**Affiliations:** 1 St Paul’s Hospital Millennium Medical College, Addis Ababa, Ethiopia; 2 University of Gondar, Gondar, Ethiopia; 3 Addis Ababa University, Addis Ababa, Ethiopia; 4 Jimma University, Jimma, Ethiopia; 5 Bill and Melinda Gates Foundation, Seattle, WA, USA; 6 RTI International, Durham, NC, USA; 7 Columbia University, New York, NY, USA

**Keywords:** dexamethasone, preterm birth, neonatal mortality, neonatal morbidity

## Abstract

*Background.* Administration of antenatal corticosteroids to pregnant mothers is one of the most effective interventions to decrease preterm neonatal mortality. In this study we assessed antenatal steroid utilization by the mother and its effect on preterm babies. *Method.* Two years prospective, multicenter, observational study was conducted in selected hospitals of Ethiopia. Significance of the study outcomes was tested by chi-square and binary logistic regression. *Result.* Out of 4919 participants, 1575 preterm babies whose gestational ages were below 35 weeks were included in the study. Use of antenatal dexamethasone was 37.5% among study participants. The risk of early onset neonatal sepsis 235 (40.4%) was higher in preterm babies whose mother took antenatal dexamethasone (*P*-value .002) than those who did not. *Conclusion.* Antenatal dexamethasone use in our study was comparable with other low and middle-income countries. Risk of early onset neonatal sepsis was higher among infants whose mother took antenatal dexamethasone.

## Background

Globally, every year an estimated 15 million infants are born prematurely (before 37 complete weeks of gestation), and this number is increasing. Every year, close to 2.5 million infants die before 28 days of life, with an average neonatal mortality rate of 18 deaths per 1000 live births. Preterm birth is a major cause of neonatal mortality. Currently, neonatal death is the most common cause of death of children under 5 years old.^1-4^ It is estimated that more than 60% of the world’s preterm births occur in sub-Saharan African and south Asian countries. Preterm birth can be associated with a number of lethal complications, particularly for those born at earlier gestational ages. Out of 5.9 million under 5 neonatal deaths, 1 million deaths are due to complications of preterm birth. In high-income countries, most babies that are >25 weeks’ gestation are able to survive. On the other hand, in low-income countries, even moderately preterm babies have high mortality rates.^2,5,6^

Administration of antenatal corticosteroids(ACS) to pregnant women who are likely to give birth preterm is found to be one of the most effective interventions to decrease preterm neonatal mortality.^7-9^ The coverage of antenatal steroids in the majority of middle- and low-income countries remain very low as compare to the high income countries.^4^ ACS use has demonstrated a 34% reduction in the incidence of respiratory distress syndrome (RDS), a 46% reduction in intraventric-ular hemorrhage, and a 31% reduction in neonatal mortality.^4,10^ Trials on administration of ACS use for preterm births in developing countries have shown mixed results with some study results comparable to those in developed countries,^11-14^ and other studies done in low resource settings on glucocorticoids did not reduce mortality and unexpectedly increase the incidence of neonatal death.^15^ The recent trials done in low resource countries showed significantly lower risks of neonatal death alone and still birth or neonatal death among those mothers who used ACS.^16^

The U.S. National Institutes of Health has recommended that for mothers with a gestational age between 24 and 34 weeks, use of ACS may improve the outcome of preterm infants, and, in particular, reduce the incidence and severity of RDS. Recommendations for ACS treatment consist of 2 doses of 12 mg of betamethasone given intramuscularly 24 hours apart, or 4 doses of 6 mg of dexamethasone given intramuscularly 12 hours apart.^8,9^

This study aims to describe the ACS utilization and its association with preterm-related complications among mothers who gave birth before 35 weeks of gestational age in 5 selected hospitals of Ethiopia. In Ethiopia, the practice is to give 4 doses of antenatal dexamethasone administered over 48 hours for those women likely to give birth between 24 and 34 weeks.^8^ Thus, the aim of this study was to describe the use of ACS among preterm births and to see its effect on preterm related complications and death in 5 selected hospitals of Ethiopia.

## Methods

The main study was a prospective, multicenter, cross-sectional, observational clinical study conducted in 5 teaching hospitals in Ethiopia for a period of nearly 2 years. The study area includes different regions of Ethiopia: Tikur Anbessa Specialized hospital, St. Paul’s Hospital and Gandhi Memorial Hospital, which are located in Addis Ababa, the capital city of Ethiopia, Gondar University Hospital which is located in northern Ethiopia, and Jimma University Hospital which is located in southwest Ethiopia. All the study hospitals are teaching hospitals where pediatricians, pediatric residents and medical interns practice. In all the study hospitals there is maternal care including high risk mother follow-up, delivery service (40 440/year) cesarean sections (25%-34% of total delivery) and blood transfusion and also neonatal care including preterm NICU admission services (2760/year), CPAP which is locally-made bubble CPAP, Kangaroo Mother Care, high risk infant follow-up clinics but there is no mechanical ventilator and parenteral nutrition support for preterm infants at the time of the study period. Data were collected on socioeconomic status, obstetric history, clinical conditions, and laboratory and imaging studies. The study protocol was published earlier.^17^

The primary study was a prospective, cross-sectional, observational study that was conducted to identify major causes of death among preterm babies in 5 selected hospitals of Ethiopia. This is a secondary study that aimed to assess the use of dexamethasone and its effect on preterm births whose gestational age was below 35 weeks.

Maternal ACS utilization was assessed among those neonates whose gestational age was below 35 weeks, based on the available medical records. When available, the number of doses was recorded. Gestational age was determined by using a hierarchy of 3 methods: ultrasound before 28 weeks of gestation when available, mothers’ report on her last menstrual period when judged reliable, and New Ballard Score. Criteria were developed to decide the best gestational age to define whether the preterm infant met the criteria of a gestational age below 35 weeks.^17^

### Operational Definitions

*Respiratory distress syndrome (RDS)* was defined as clinical symptoms of respiratory distress in preterm babies including (tachypnea, subcostal or intercostal retraction, nasal flaring, grunting, or cyanosis, bilateral decrease air entry) requiring oxygen supplement. Chest X-ray was not done for all neonates with impression of RDS, so diagnosis of RDS mainly rely on clinician’s decision.

*Early onset neonatal sepsis (EONs)* was made (based on clinical sighs including temperature instability, respiratory distress, decreased gestational age appropriate neonatal reflexes, altered mentation, abnormal body movement, or poor feeding) in the first 72 hours of life. Neonates often underwent laboratory investigations including complete blood count, blood culture-C-reactive protein, and CSF analysis and culture to establish the diagnosis sepsis.

*Intraventricular hemorrhage (IVH)* was defined and graded by trans-fontanel ultrasound examination.

*Necrotizing Enterocolitis (NEC)* was defined as neonates who presented with abdominal distension and hematochezia and showed pneumatosis intestinalis on abdominal radiograph. In all cases the final diagnosis made by the treating physician was used. Mortality for this study was defined as a neonatal death at or before 28 days of life.^17^

*Study participants*: The study was conducted in 5 selected hospital of Ethiopia. All preterm babies whose gestational age was below 35 weeks were included in the study.

## Eligibility for Steroid Utilization

All live-born preterm neonates whose gestational age was below 35 weeks and whose mother gave consent for study participation were included in the study.

## Exclusion Criteria

We excluded infants with a gestational age below 24 completed weeks and above 34 completed weeks, mothers with a diagnosis of chorioamnionitis, and preterm babies who were not admitted to one of the study’s neonatal intensive care units (NICU). Also excluded were mothers/infants with missing data or unknown ACS utilization status.

## Quality Assurance

All preterm infants admitted to the study hospital were evaluated twice daily by a research nurse who documented the findings as per study forms. A supervisor checked to ensure all forms were complete. Also, the research nurse confirmed that samples for laboratory evaluations were taken in sufficient quantity. Relevant laboratory and radiology information were reviewed by the pathology, radiology and laboratory personnel respectively. The investigators conducted on spot visits to each hospital regularly to ensure the data quality and provide support and troubleshooting as necessary.

## Data Entry and Statistical Analysis

Data were double entered into a computer using the data management system developed for the study. Data were transferred on a weekly basis from each NICU to the data center at Addis Ababa University, creating a complete data repository. Data were merged to 1 master data set for cleaning and further analysis. Descriptive statistics were performed to compare the overall distribution of antenatal dexamethasone utilization versus the socio-demographic status of study participant using SPSS (version-20.0). Chi-square tests, odds ratios, and a *P*-value with 95% CI were used to test the association of dexamethasone utilization versus outcomes.

## Ethical Approval and Consent to Participate

The study protocol was approved by the ethical committees of all participating hospitals and the ethics review committee at Addis Ababa University (approval letter MF03-008). Consent was requested from women and their families, as appropriate, in their local language. All women provided informed consent prior to participation in the study. Confidentiality of the information was strictly maintained.

## Results

**Figure 1.**
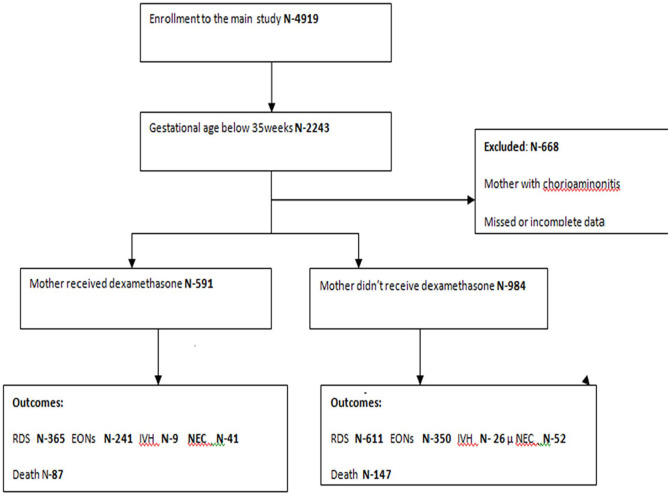
Enrollment flow diagram. Abbreviations: RDS: respiratory distress syndrome; IVH, intraventricular hemorrhage; NEC, necrotizing enterocolitis, EONs, early onset neonatal sepsis.

Among the 4919 enrolled preterm babies, 2243 had a gestational age below 35 weeks. Of these 2243 preterm births, we excluded 668 babies whose records had incomplete information or whose mothers had chorioamnionitis during pregnancy. Of these, 1575 had complete information and were included in the study (Figure 1).Of these, 591 (37.5%) of the preterm babies’ mothers received antenatal dexamethasone.

Among the mothers who received dexamethasone, 228 (39.2%) were 25 to 29 years of age and 269 (39%) of the mothers give birth at gestational ages between 28 and 32 weeks. Statistically significant association was seen between educational status of the mothers and antenatal dexamethasone utilization where half of the mothers 103 (50%) who had higher formal education received antenatal dexamethasone, and only one-third 177 (33.1%) of mothers who had primary education received dexamethasone. Among mothers who received antenatal dexamethasone, 29 (25.2%) had no antenatal care and 360 (37.5%) and 187 (42.4%) of the mothers respectively had less than 4 and 4 or more antenatal care visits before delivery. About 581 (44.4%) of mothers who give birth at the hospital received ACS, whereas only 5 (2.6%) who give birth at health center received ACS (Figure 1 and Table 1).

**Table 1.** Maternal Socio-Demographic Data on Trends and Effects of Antenatal Dexamethasone on Preterm Related Complications and Outcomes in 5 Selected Hospitals of Ethiopia.

Characteristics	Steroid administered		*P*-value
	Yes (%)	No (%)	
Age of the mother			
Less than 20	28 (26.4)	78 (73.6)	.100
20-24 years	161 (36.4)	281 (63.6)	
25-29 years	228 (39.2)	356 (60.8)	
30-34 years	116 (41.1)	166 (58.9)	
≥35 years	59 (37.1)	101 (62.9)	
Formal education			
None/not able to read	125 (34.7)	235 (65.3)	.001
None/able to read	41 (39.0)	64 (61.0)	
Primary	177 (33.1)	357 (66.9)	
Secondary	133 (38.0)	217 (62.0)	
Higher education	103 (50.0)	103 (50.0)	
Gestational age			
Less than 28 weeks	23 (31.1)	51 (68.9)	.340
28-31 weeks	269 (39.0)	420 (61.0)	
32-34 weeks	299 (36.8)	513 (33.2)	
ANC visits			
None	29 (25.2)	86 (74.8)	.002
Less than 4	360 (37.5)	603 (62.5)	
Greater or equal to 4	187 (42.4)	254 (57.6)	
Place of delivery			
Hospital	581 (44.6)	723 (55.4)	.000
Health center	5 (2.6)	188 (97.4)	
Home	0	45 (100)	
Other^a^	0	14 (100)	

Abbreviation: ANC, antenatal care.

a Other—delivery on the way to hospital in the ambulance.

From a total 1575 cases enrolled in this sub-study, 591 (37.5%) of the mothers received dexamethasone before delivery. Four or more doses of dexamethasone were given to 287 (58.0%) of the mothers who received steroids and in close to half 108 (47.0%), the duration from the last dose of dexamethasone until delivery was less than 24 hours. Among all preterm neonates whose gestational age was below 35 weeks, the following complications were recorded: RDS 972 (61.75), IVH 38(2.4%), NEC 95 (6.0%), EONS 590 (37.3), and 234 (14.7%) died before 28 days of life (Table 2).

**Table 2.** Antenatal Dexamethasone Use and Number of Doses by Mothers of Preterm Infants in 5 Selected Hospitals of Ethiopia.

Characteristics	Number (%)
Steroids	
Yes	591 (37.5)
No	984 (62.5)
Total	1575 (100)
If yes, number of steroid doses	
1 dose	108 (21.9)
2 doses	79 (16.0)
3 doses	18 (3.7)
≥4 doses	287 (58.0)
Total	493 (100)
Duration from last steroid dose before birth	
Less than 24 hours	108 (47.0)
24-47 hours	25 (10.9)
48 hours-7 days	93 (40.4)
Greater than 7 days	4 (1.7)
Total	230 (100)
Outcomes	
RDS	972 (61.7)
IVH	38 (2.4)
NEC	95 (6.0)
EONs	590 (37.3)
Death	234 (14.7)

Abbreviations: RDS, respiratory distress syndrome; IVH, intraventricular hemorrhage; NEC, necrotizing enterocolitis; EONs, early onset neonatal sepsis.

The risk of RDS,IVH,NEC and death in preterm babies’ whose gestational age was below 35 weeks who gave birth in the hospital did not differ significantly whether the mothers took antenatal dexamethasone or not (60.7%, 1.5%, 6.9%, and 14.7% vs 65.0%, 2.5%, 4.7%, and 14.9%, respectively).The risk of EONs was higher among preterm babies whose mother took antenatal dexamethasone (40.4%) compared to those who did not take dexamethasone (32.1%) (*P*-value .002 and odds ratio 0.696; Table 3).

**Table 3.** Effect of Antenatal Dexamethasone Administration on Preterm Babies’ Outcomes Among Those Delivered in the Hospitals.

Outcomes	Infants with dexamethasone administered				
	Yes N (%)	No N (%)	Total N (%)	Odds ratio (95% CI)	*P*-value
RDS	353 (60.7)	470 (65.0)	823 (100)	1.2 (0.957-0.504)	.114
IVH	9 (1.5)	18 (2.5)	27 (100)	1.623 (0.724-3.639)	.236
NEC	40 (6.9)	34 (4.7)	74 (100)	0.66 (0.417-1.069)	.091
EONs	235 (40.4)	232 (32.1)	467 (100)	0.696 (0.554-0.874)	.002
Death	87 (14.7)	147 (14.9)	234 (100)	1.017 (0.763-1.356)	.906

Abbreviations: RDS, respiratory distress syndrome, IVH, intraventricular hemorrhage, NEC, necrotizing enterocolitis; EONs, early onset neonatal sepsis.

The risk of RDS, IVH, NEC, and death did not show any difference among preterm infants whose mothers received single or multiple doses of antenatal dexamethasone. But the risk of having early onset sepsis was higher in those who received a single dose compared to multiple doses (53.0% vs 39.7%) with odds ratio of 1.714 (1.100-2.671) and *P*-value of 0.017 (Table 4).

**Table 4.** Complications and Mortality in the First 28 days of Life Among Those Infants Whose Mothers Received Different Doses of Antenatal Dexamethasone in 5 Selected Hospitals of Ethiopia.

Characteristics	Doses of dexamethasone		Odds ratio (crud) with 95% CI	*P*-value
	1 dose N (%)	>1 doses N (%)		
RDS Yes	56 (56.0)	234 (61.9)	0.783 (0.501-1.224)	.282
No	44 (44.0)	144 (38.1)		
Total	100 (100)	378 (100)		
EONs Yes	53 (53.0)	150 (39.7)	1.714 (1.100-2.671)	.017
No	47 (47.0)	228 (60.3)		
Total	100 (100)	378 (100)		
IVH Yes	0 ()	9 (2.4)	0.00	.119
No	100 (100)	369 (97.6)		
Total	100 (100)	378 (100)		
NEC Yes	9 (9.0)	25 (6.6)	1.396 (0.630-3.095)	.409
No	91 (91.0)	353 (93.4)		
Total	100 (100)	378 (100)		
Death Yes	10 (10.0)	60 (15.9)	0.589 (0.290-1.197)	.140
No	90 (90.0)	318 (84.1)		
Total	108 (100)	378 (100)		

Abbreviations: RDS, respiratory distress syndrome; EONs, early onset neonatal sepsis, IVH, intraventricular hemorrhage, NEC, necrotizing enterocolitis.

## Discussion

In high resource settings, ACS have been shown to improve the survival rate of preterm neonates and to reduce the incidence of preterm related complications such as RDS, IVH, NEC, and sepsis. The WHO and Ethiopian Ministry of Health have recommended ACS administration for women in anticipated preterm labor who have good gestational age dating, unless the mother has chorioamnionitis.^8,9^ To date, there have not been published reports of the prevalence of ACS utilization in Ethiopia. This study provides a baseline for further studies. The study covers the use of ACS and its effect on preterm babies’ outcome in 5 selected hospitals of Ethiopia. Our major findings were that the use of antenatal dexamethasone among women with a preterm delivery at <35 weeks gestation was 37.5%. 21.9% received only 1 dose and 58.0% of the mothers received the recommended 4 or more doses of dexamethasone. Few of the mothers who gave birth at a health center received antenatal dexamethasone whereas over 44%who gave birth at a hospital received dexamethasone. While this was not a randomized trial, we did not find significant differences in RDS, NEC, or IVH based on whether the mother took antenatal dexamethasone or not. The risk of EONS was higher among infants whose mother took antenatal dexamethasone. This finding is consistent with the Global Network study that also found an increased risk of infection among infants exposed to ACS prenatally.^15^

The use of ACS in our study was comparable reports from other low and middle-income countries. In 1 study in 4 Southeast Asian countries, 40% of mothers received ACS prior to preterm delivery.^1^ In other studies conducted in low and middle-income countries including deliveries conducted in the community at health facilities and in hospitals, the prevalence of ACS utilization ranged from 4% to 45%.^18,19^ But the use of ACS in our study was far below the rates for developed countries where the coverage is often reported at more than 90%.^4^

The risk of having EONs was higher (40.4% vs 32.1%) among preterm babies whose mother took antenatal dexamethasone (odds ratio 0.696 (0.554-0.874) and *P*-value 0.002). There was not a statistically significant difference seen on the risk of having IVH, NEC, or death with ACS use. Similarly, a retrospective study done in South Africa showed no significant difference in the risk of RDS and NEC with ACS use.^20^ Similar to our study, a 10 years retrospective study in Taiwan showed no statistical difference in the incidence of IVH, NEC, and sepsis with ACS use.^21^ In mostly high income countries, ACS administration was associated with a decrease the incidence of RDS, IVH, and sepsis in the first 48 hours of life by 34%, 46%, and 43% respectively in a high-quality systematic review including 18 trials.^4^

In our study, there was no difference on neonatal mortality in the first 28 days of life among those mothers who received or did not receive ACS (14.7% vs 14.9%). The Global Network’s study also did not find improved survival among those with exposure to ACS.^15^ But other studies in low and middle-income countries have shown significant survival improvement among those infants whose mothers received ACS (58.2%) compared to those who did not receive ACS (32.0%).^13,19^ A high-quality systemic review in developed countries also showed that mortality was reduced by 31%.^4^

## Strength

The study was a large multi-centric population-based study conducted in different parts of Ethiopia. Data collection was well structured and quality assurance of the data was also checked periodically.

## Limitation

This study is part of a project on major causes of death in preterm babies.^17,22,23^ The study was an observational study, and because it was not a randomized trial, results must be interpreted with caution.

## Conclusion

Antenatal dexamethasone use in our study was comparable with other low- and middle-income countries. Risk of early onset neonatal sepsis was higher among infants whose mother took antenatal dexamethasone.
